# Supplementary material for: On the use of discrete-time quantum walks in decision theory
Source: PLoS One. 2022 Aug 30;17(8):e0273551. doi: 10.1371/journal.pone.0273551 (PMC9426940; doi:10.1371/journal.pone.0273551)
Supplement: S1 Appendix — (PDF) [file pone.0273551.s001.pdf]

Mathematical foundation of quantum decision theory

The following appendix presents the mathematical structure of QDT, first in the general case and then focusing on binary choices. For a more complete treatment, the reader is referred to [1–3]. Some of the equations presented in the main text are reported also here for convenience.

S1.1 General case: choice between N alternatives

In this section, we sketch the derivation to obtain choice probabilities (probabilities of composite events) in QDT, in the case of N alternatives. Denote with C_n an operationally testable event (choice), indexed by $n \in \{1, ..., N\}$, and with $I = \{I_\alpha\}$ a set of inconclusive events (subconscious deliberations), labeled by α. The corresponding space of events is an Hilbert Space $\mathcal{H}$, given by

$$\begin{gathered} \mathcal{H} = \mathcal{H}_C \otimes \mathcal{H}_I \\ \mathcal{H}_C = \mathrm{Span}\{|n\rangle\} \;\; \mathcal{H}_I = \mathrm{Span}\{|\alpha\rangle\} \end{gathered} \tag{1}$$

where $|n\rangle$ (resp. $|\alpha\rangle$) is the state associated with event C_n (resp. I_α). We also associate to the set of inconclusive events I the state $|I\rangle$

$$|I\rangle = \sum_\alpha b_\alpha |\alpha\rangle \tag{2}$$

where $\{b_\alpha\}$ are random complex numbers. A decision maker is assumed to be characterized by a statistical quantum operator $\hat{\rho}$, called strategic state of mind. A composite event, henceforth termed prospect, is written as

$$\pi_n = C_n \otimes I \tag{3}$$

with the corresponding prospect state and prospect operator

$$\begin{gathered} |\pi_n\rangle = |n\rangle \otimes |I\rangle = \sum_\alpha b_\alpha |n\alpha\rangle \\ \hat{P}(\pi_n) = |\pi_n\rangle \langle \pi_n| \end{gathered} \tag{4}$$

The prospect probability is

$$p(\pi_n) = Tr_{\mathcal{H}} \hat{\rho} \hat{P}(\pi_n) \tag{5}$$

where $Tr_{\mathcal{H}}$ denotes the trace operation over Hilbert space $\mathcal{H}$. The prospect probabilities are subjected to the normalization constraint

$$\sum_n p(\pi_n) = 1, \;\; 0 \le p(\pi_n) \le 1 \tag{6}$$

By simple calculations, $p(\pi_n)$ is written as a sum of (positive-defined) diagonal and (sign-undefined) off-diagonal terms

$$\begin{aligned} p(\pi_n) &= f(\pi_n) + q(\pi_n) \\ f(\pi_n) &= \sum_\alpha |b_\alpha|^2 \langle n\alpha| \hat{\rho} |n\alpha\rangle \\ q(\pi_n) &= \sum_{\alpha \neq \alpha'} b^*_\alpha b_{\alpha'} \langle n\alpha| \hat{\rho} |n\alpha'\rangle \end{aligned} \tag{7}$$

As reported in the main text, QDT imposes a quantum-classical correspondence similar to the principle developed in Physics [4], which requires that classical theory is a particular case of quantum theory, implying in our context

$$\lim_{q(\pi_n)\to 0} p(\pi_n) = f(\pi_n) \tag{8}$$

From Eq. 8, we see that the term f plays the role of the classical probability, and it has to be normalized

$$\sum_n f(\pi_n) = 1 \qquad 0 \le f(\pi_n) \le 1 \tag{9}$$

From the normalization of the prospect probabilities $\{p(\pi_n)\}$ (Eq. 6) and normalization of utility factors $\{f(\pi_n)\}$ (Eq. 9), we get the following properties [5]

$$\begin{aligned} -1 \le q(\pi_n) \le 1 \\ \sum_n q(\pi_n) = 0 \quad \text{(alternation law)} \end{aligned} \tag{10}$$

S1.2 QDT for binary choices

In this section, we apply QDT to a binary decision task between lottery A and B. We introduce two observables: the choice $C = \{A, B\}$, represented by a quantum operator $\hat{C}$, and $I = \{I_1, I_2\}$, represented by operator $\hat{I}$. The operators $\hat{C}$ and $\hat{I}$ act on Hilbert spaces $\mathcal{H}_C = \text{span}\{|A\rangle, |B\rangle\}$ and $\mathcal{H}_I = \text{span}\{|I_1\rangle, |I_2\rangle\}$, respectively. Observable C takes value A (resp. B) when lottery A (resp. B) is chosen. As suggested by Yukalov and Sornette [6], observable I embodies: i) the uncertainty caused by the subjective hesitations of the decision maker with respect to his/her correct understanding of the problem and his/her knowledge of what would be the best criterion for making a particular choice; ii) the doubt about the objectivity of the setup suggesting the choice. Thus I_1 represents the confidence of the decision maker in the empirical setup as well as in the correctness of his/her decision. Conversely, I_2 corresponds to the disbelief of the decision maker in the suggested setup and/or in his/her understanding of the appropriate criteria for the choice.

The state of mind, characterizing the personal attributes of a decision maker at a given time, is defined in the tensor-product space

$$\mathcal{H} = \mathcal{H}_C \otimes \mathcal{H}_I \tag{11}$$

and is written as[1]

$$|\psi\rangle = c_{A_1} |AI_1\rangle + c_{A_2} |AI_2\rangle + c_{B_1} |BI_1\rangle + c_{B_2} |BI_2\rangle \tag{12}$$

To allow the possible emergence of quantum interference, we assume that lotteries A and B correspond to *composite* prospect states of the form:

$$\begin{cases} |\pi_A\rangle = \alpha |A\rangle \otimes (\gamma_{A_1} |I_1\rangle + \gamma_{A_2} |I_2\rangle) \equiv a_1 |AI_1\rangle + a_2 |AI_2\rangle \\ |\pi_B\rangle = \beta |B\rangle \otimes (\gamma_{B_1} |I_1\rangle + \gamma_{B_2} |I_2\rangle) \equiv b_1 |BI_1\rangle + b_2 |BI_2\rangle \end{cases} \tag{13}$$

The prospect states correspond to the decision maker choosing either lottery, with indefinite (unobserved) mixed feelings about the setup and context. The prospect

[1] The amplitude coefficients are in principle time-dependent, accounting for the evolution of decision maker's attitudes due to endogenous (feelings, thoughts) and exogenous (external interactions) processes. Also, the state of mind is in general a statistical operator. For the scope of this paper, we neglect time dependence.

probabilities are then obtained through the usual Born rule

$$\begin{aligned}
p(\pi_A) &= |\langle \pi_A|\psi\rangle|^2 \\
&= \sum_{k=1}^{2} |a_k|^2 \langle AI_k|\psi\rangle \langle \psi|AI_k\rangle + \sum_{k\neq k'} a_k^* a_{k'} \langle AI_k|\psi\rangle \langle \psi|AI_{k'}\rangle \\
&= |a_1 c_{A_1}|^2 + |a_2 c_{A_2}|^2 + c_{A_1}^* a_1 a_2^* c_{A_2} + c_{A_2}^* a_2 a_1^* c_{A_1} \\
&= f(\pi_A) + q(\pi_A)
\end{aligned} \tag{14}$$

where $f(\pi_A)$ (resp. $q(\pi_A)$) represents the contribution to the total probability coming from diagonal (resp. off-diagonal) terms (analogous form holds for $p(\pi_B) = 1 - p(\pi_A)$). Turning our attention to the non-diagonal term $q(\pi_n)$, its properties (Eq. 10) for a binary choice read

$$\begin{aligned}
-1 \leq q(\pi_n) \leq 1 \;\; n \in \{A, B\} \\
\sum_n q(\pi_n) = 0 \rightarrow q(\pi_A) = -q(\pi_B)
\end{aligned} \tag{15}$$

Moreover, its specific expression, say of $q(\pi_A)$, can be rewritten as

$$\begin{aligned}
q(\pi_A) &= c_{A_1}^* a_1 a_2^* c_{A_2} + c_{A_2}^* a_2 a_1^* c_{A_1} \\
&= 2Re(c_{A_1}^* a_1 a_2^* c_{A_2}) \\
&= 2|c_{A_1}^* a_1 a_2^* c_{A_2}| cos(\Delta^A)
\end{aligned} \tag{16}$$

where Δ^A is the so-called uncertainty angle [5]. Eq. 16 is actually equivalent to [7]

$$q(\pi_A) = 2f(\pi_A)\sqrt{x(1-x)} cos(\Delta^A) \tag{17}$$

for some $x \in [0, 1]$. Eq. 17 implies that $|q(\pi_A)| \leq f(\pi_A)$. An analogous derivation for $q(\pi_B)$ implies $|q(\pi_b)| \leq f(\pi_B) = 1 - f(\pi_A)$. Combining this result with the alternation law (Eq. 15) we have

$$|q(\pi_A)| = |q(\pi_B)| \leq min(f(\pi_A), f(\pi_B)) \tag{18}$$

References

1. Yukalov VI, Sornette D. Mathematical structure of quantum decision theory. Advances in Complex Systems. 2010;13(05):659–698.

2. Yukalov VI, Sornette D. Quantitative predictions in quantum decision theory. IEEE Transactions on Systems, Man, and Cybernetics: Systems. 2018;48(3):366–381.

3. Yukalov VI, Yukalova EP, Sornette D. Information processing by networks of quantum decision makers. Physica A: Statistical Mechanics and its Applications. 2018;492:747–766.

4. Bohr N. Über die Serienspektra der Elemente. Zeitschrift für Physik. 1920;2(5):423–469.

5. Yukalov VI, Sornette D. Processing information in quantum decision theory. Entropy. 2009;11(4):1073–1120.

6. Yukalov VI, Sornette D. Positive operator-valued measures in quantum decision theory. In: International Symposium on Quantum Interaction. Springer; 2014. p. 146–161.

7. Vincent S, Kovalenko T, Yukalov VI, Sornette D. Calibration of quantum decision theory, aversion to large losses and predictability of probabilistic choices. Swiss Finance Institute Research Paper 16-31. 2016;.
