# Supplementary material for: On the use of discrete-time quantum walks in decision theory
Source: PLoS One. 2022 Aug 30;17(8):e0273551. doi: 10.1371/journal.pone.0273551 (PMC9426940; doi:10.1371/journal.pone.0273551)
Supplement: S2 Appendix — (PDF) [file pone.0273551.s002.pdf]

Mathematical properties of discrete-time quantum walks

S2.1 Solution for an unbounded DTQW

We present the solution of the quantum walk as described in the main text, based on the result obtained by Ambainis et al. [1]. They study a specific type of walk called the Hadamard walk, where the Hadamard matrix H is used as the quantum coin:

$$H = \frac{1}{\sqrt{2}} \begin{pmatrix} 1 & 1 \\ 1 & -1 \end{pmatrix} \tag{1}$$

We follow the same procedure but we use the general form of the coin operator from Eq (24), together with Eq (25) (26) in the main text, and write:

$$\begin{aligned} |\psi(t)\rangle &= U |\psi(t-1)\rangle \\ &= \sum_{x\in\mathbb{Z}} |{\leftarrow}\rangle \langle{\leftarrow}| \cdot C |\psi(x,\ t-1)\rangle \otimes |x-1\rangle + |{\rightarrow}\rangle \langle{\rightarrow}| \cdot C |\psi(x,\ t-1)\rangle \otimes |x+1\rangle \\ &= \sum_{x\in\mathbb{Z}} M_- |\psi(x,\ t-1)\rangle \otimes |x-1\rangle + M_+ |\psi(x,\ t-1)\rangle \otimes |x+1\rangle \end{aligned} \tag{2}$$

Here we write $|{\leftarrow}\rangle \langle{\leftarrow}| \cdot C$ as M_- and $|{\rightarrow}\rangle \langle{\rightarrow}| \cdot C$ as M_+. Thus, we have:

$$|\psi(x,t)\rangle = M_- |\psi(x+1,\ t-1)\rangle + M_+ |\psi(x-1,\ t-1)\rangle \tag{3}$$

We take the discrete Fourier transform to get the momentum state at time t:

$$\begin{aligned} |\widetilde{\psi}(k,t)\rangle &= \sum_{x\in\mathbb{Z}} |\psi(x,\ t)\rangle e^{ikx} \\ &= \sum_{x\in\mathbb{Z}} \left(M_- |\psi(x+1,\ t-1)\rangle + M_+ |psi(x-1,\ t-1)\rangle\right) e^{ikx} \\ &= e^{-ik} M_- \sum_{x\in\mathbb{Z}} |\psi(x+1,\ t-1)\rangle e^{ik(x+1)} + e^{ik} M_+ \sum_{x\in\mathbb{Z}} |\psi(x-1,\ t-1)\rangle e^{ik(x-1)} \\ &= \left(M_- e^{-ik} + M_+ e^{ik}\right) |\widetilde{\psi}(k,t-1)\rangle \\ &= M_k |\widetilde{\psi}(k,t-1)\rangle \end{aligned} \tag{4}$$

Therefore, $|\widetilde{\psi}(k,t)\rangle = M_k^t |\widetilde{\psi}(k,0)\rangle$. The next step is to find the eigen-decomposition of M_k, so that we can obtain M_k^t with:

$$M_k^t = \left(\lambda_k^-\right)^t |v_k^-\rangle \langle v_k^-| + \left(\lambda_k^+\right)^t |v_k^+\rangle \langle v_k^+| \tag{5}$$

Here $\lambda_k^\pm$ are two eigenvalues and $|v_k^\pm\rangle$ are the corresponding eigenvectors. Without loss of generality, we write the spin state $|{\leftarrow}\rangle$ as $[1,\ 0]^T$ and $|{\rightarrow}\rangle$ as $[0,\ 1]^T$, so we have:

$$\begin{aligned} M_- &= |{\leftarrow}\rangle \langle{\leftarrow}| \cdot C = \begin{pmatrix} e^{i\xi}\sqrt{\rho} & e^{i\zeta}\sqrt{1-\rho} \\ 0 & 0 \end{pmatrix} \\ M_+ &= |{\rightarrow}\rangle \langle{\rightarrow}| \cdot C = \begin{pmatrix} 0 & 0 \\ e^{-i\zeta}\sqrt{1-\rho} & -e^{-i\xi}\sqrt{\rho} \end{pmatrix} \end{aligned} \tag{6}$$

Hence:

$$M_k = \begin{pmatrix} e^{i(\xi-k)}\sqrt{\rho} & e^{i(\zeta-k)}\sqrt{1-\rho} \\ e^{-i(\zeta-k)}\sqrt{1-\rho} & -e^{-i(\xi-k)}\sqrt{\rho} \end{pmatrix} \tag{7}$$

Following the standard procedure of eigen-decomposition, we obtain:

$$\lambda_k^{\pm} = -i\sqrt{\rho}\sin(\xi-k) \pm \sqrt{1-\rho\sin^2(\xi-k)} \tag{8}$$

Since M_k is unitary, we can write $\lambda_k^{\pm} = e^{-i\omega_k^{\pm}}$, where:

$$\begin{gathered} \sin\left(\omega_k^{+}\right) = \sqrt{\rho}\sin(\xi-k) \\ \omega_k^{-} = \pi - \omega_k^{+} \end{gathered} \tag{9}$$

The eigenvectors are given by:

$$\begin{aligned} |v_k^{-}\rangle &= \frac{1}{N_k^{-}}\begin{pmatrix} \sqrt{1-\rho} \\ -e^{i\left(\omega_k^{-}-\zeta+k\right)} - e^{i(\xi-\zeta)}\sqrt{\rho} \end{pmatrix} \\ |v_k^{+}\rangle &= \frac{1}{N_k^{+}}\begin{pmatrix} \sqrt{1-\rho} \\ e^{-i\left(\omega_k^{+}+\zeta-k\right)} - e^{i(\xi-\zeta)}\sqrt{\rho} \end{pmatrix} \end{aligned} \tag{10}$$

where $N_k^{\pm}$ are the normalization factors:

$$N_k^{\pm} = 2 \mp 2\sqrt{\rho}\cos k \mp \omega_k^{\pm} - \xi \tag{11}$$

Imposing the results of Eq 9, 10 to Eq 3, 4, we now have:

$$|\widetilde{\psi}(k,t)\rangle = \left(e^{i\omega_k^{-}t}\,|v_k^{-}\rangle\langle v_k^{-}| + e^{i\omega_k^{+}t}\,|v_k^{+}\rangle\langle v_k^{+}|\right)|\widetilde{\psi}(k,0)\rangle \tag{12}$$

$|\widetilde{\psi}(k,0)\rangle$ can be obtained from the initial state at position x by Fourier transform given in the first line of (4). We use a continuous inverse Fourier transform for $|\widetilde{\psi}(k,t)\rangle$, because for unbounded walks there is a continuum of k, such that:

$$|\psi(x,t)\rangle = \frac{1}{2\pi}\int_{-\pi}^{\pi}|\widetilde{\psi}(k,t)\rangle\, e^{-ikx}dk \tag{13}$$

Then, the time-dependent solution $|\psi(t)\rangle$ for the state of a quantum walk is obtained from Eq (23) in the main text.

S2.2 Parameters controlling the evolution of quantum walks

Consider the simple initial state

$$|\psi(t=0)\rangle = \begin{bmatrix} L_0 \\ R_0 \end{bmatrix} \otimes |0\rangle = \begin{bmatrix} e^{i\alpha}\sqrt{\eta} \\ e^{i\beta}\sqrt{1-\eta} \end{bmatrix} \otimes |0\rangle \tag{14}$$

We parametrize the components of the spin state with α, β and η to give the most general form of the initial state, which satisfies the normalization condition $|L_0|^2 + |R_0|^2 = 1$. From Section "S2.1", we get:

$$|\widetilde{\psi}(k,0)\rangle = \begin{bmatrix} e^{i\alpha}\sqrt{\eta} \\ e^{i\beta}\sqrt{1-\eta} \end{bmatrix} \otimes |k\rangle \tag{15}$$

We can then write the coefficient of the eigenvectors $|v_k^{\pm}\rangle$ as:

$$e^{i\omega_k^{\pm}t}\langle v_k^{\pm}|\widetilde{\psi}(k,0)\rangle = \frac{e^{i\omega_k^{\pm}t+\alpha}}{N_k^{\pm}}\left(\sqrt{\eta(1-\rho)} + \sqrt{1-\eta}\,e^{i(\beta-\alpha+\zeta)}\left(e^{i(\mp\omega_k^{\pm}-k} - e^{-i\xi}\sqrt{\rho}\right)\right) \tag{16}$$

The global phase $i\omega_k^{\pm} t + \alpha$ does not contribute to the probability distribution in either the spin or the position space. In the rest of the equation, η and ρ have different effects on the distribution. α and β only occur in combination with ζ. Therefore, if we keep two of them fixed, say α, β, and vary ζ, we can obtain all the possible evolutions of the probability distribution. Further, for a one step evolution, according to Eq (28) in the main text, a wave packet $\left(\psi_L(x,\ t),\ \psi_R(x,\ t)\right)^T$ is split into two wave packets $\left(\psi_L(x-1,\ t+1),\ 0\right)^T$ and $\left(0,\ \psi_R(x+1,\ t+1)\right)^T$ with magnitudes

$$\begin{aligned} \left|\psi_L(x-1,\ t+1)\right|^2 =& \rho\left|\psi_L(x,\ t)\right|^2 + (1-\rho)\left|\psi_R(x,\ t)\right|^2 \\ &+ 2\sqrt{\rho(1-\rho)} Re\left(e^{i(\xi-\zeta)}\psi_L(x,\ t)\psi_R^*(x,\ t)\right) \\ \left|\psi_R(x+1,\ t+1)\right|^2 =& (1-\rho)\left|\psi_L(x,\ t)\right|^2 + \rho\left|\psi_R(x,\ t)\right|^2 \\ &- 2\sqrt{\rho(1-\rho)} Re\left(e^{i(\xi-\zeta)}\psi_L(x,\ t)\psi_R^*(x,\ t)\right) \end{aligned} \tag{17}$$

where ψ_R^* is the complex conjugate of ψ_R. Note that $\xi-\zeta$ act together to influence the evolution at each time step. So we can keep ζ fixed at 0 and only vary ξ.

S2.3 Dependence of var_p, $bias_p$ and $bias_s$ on different parameters

By assigning a value 0 to the left spin and a value 1 to the right spin (the value assigned does not affect the result), we can define the following:

$$\begin{aligned} var_p &= \sum_{x\in\mathbb{Z}} \left(x-\mu_p\right)^2 p(x) \\ bias_p &= \frac{\sum_{x\in\mathbb{Z}} \left(x-\mu_p\right)^3 p(x)}{\left[\sum_{x\in\mathbb{Z}} \left(x-\mu_p\right)^2 p(x)\right]^{3/2}} \\ bias_s &= \frac{\left(0-\mu_s\right)^3 p(L) + \left(1-\mu_s\right)^3 p(R)}{\left[\left(0-\mu_s\right)^2 p(L) + \left(1-\mu_s\right)^2 p(R)\right]^{3/2}} \end{aligned} \tag{18}$$

where var_p and $bias_p$ are the variance and bias (or skewness) of the distribution in the position space, $bias_s$ is the bias of distribution in the spin space. μ_p and μ_s are the mean in position and in the spin space, which can be expressed as:

$$\begin{aligned} \mu_p &= \sum_{x\in\mathbb{Z}} x p(x) \\ \mu_s &= 0\cdot p(L) + 1\cdot p(R) \end{aligned} \tag{19}$$

References

1. Ambainis A, Bach E, Nayak A, Vishwanath A, Watrous J. One-dimensional quantum walks. In: Proceedings of the thirty-third annual ACM symposium on Theory of computing; 2001. p. 37–49.

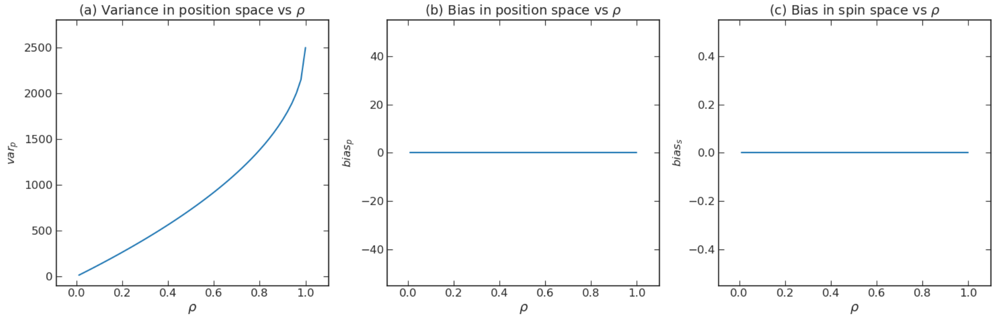

Fig 1. var_p, $bias_p$ and $bias_s$ vs ρ with $\xi = 0$, $\eta = 0.5$ at $t = 100$.

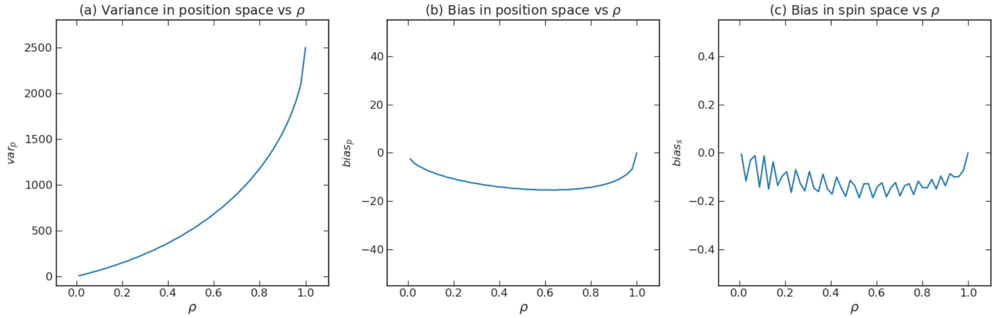

Fig 2. var_p, $bias_p$ and $bias_s$ vs ρ with $\xi = \pi/2$, $\eta = 0.5$ at $t = 100$.

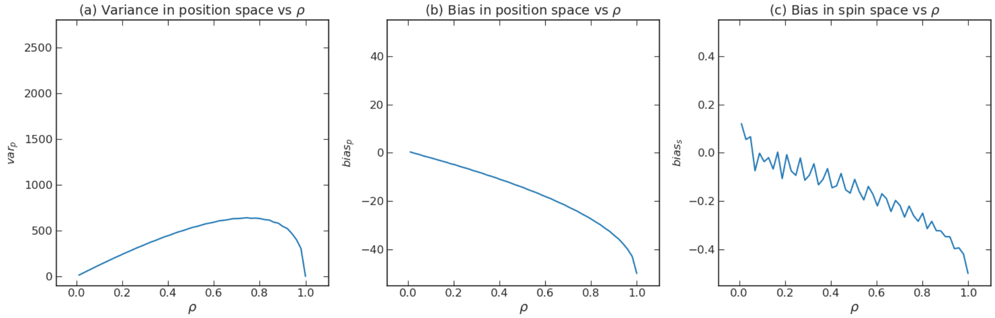

Fig 3. var_p, $bias_p$ and $bias_s$ vs ρ with $\xi = 0$, $\eta = 1$ at $t = 100$.

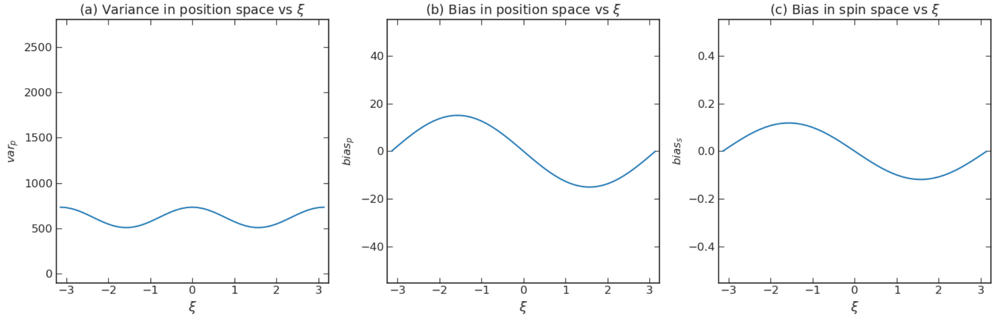

Fig 4. var_p, $bias_p$ and $bias_s$ vs ξ with $\rho = 0.5$, $\eta = 0.5$ at $t = 100$

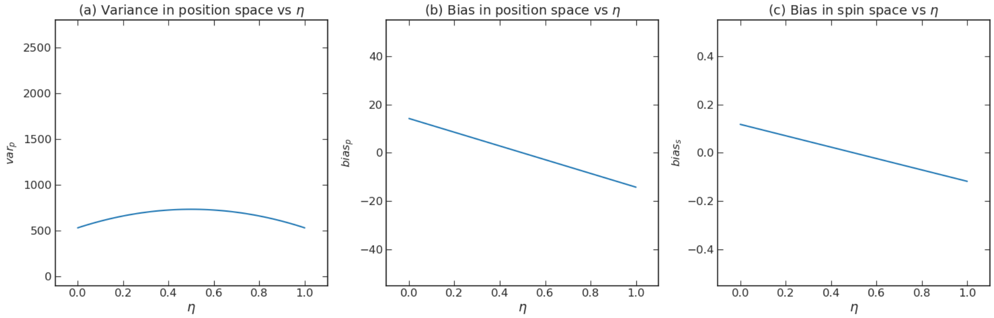

Fig 5. var_p, $bias_p$ and $bias_s$ vs η with $\rho = 0.5$, $\xi = 0$ at $t = 100$
