# Supplementary material for: On the use of discrete-time quantum walks in decision theory
Source: PLoS One. 2022 Aug 30;17(8):e0273551. doi: 10.1371/journal.pone.0273551 (PMC9426940; doi:10.1371/journal.pone.0273551)
Supplement: S3 Appendix — (PDF) [file pone.0273551.s003.pdf]

An alternative way of encoding objective probabilistic information

The second possibility for encoding an objective probability employs η in a simple initial state $\left(\sqrt{\eta},\ i\sqrt{1-\eta}\right)^T \otimes |x_0\rangle$ as the objective probability p for an event to occur (and $1-\eta$ is the probability that the event does not occur). The distances from the starting position x_0 to the left end $x = 0$ and the right end $x = N$ are equal. The conditional absorbing probability $\pi(t)$ at the left end represents the subjective probability, the same as Eq (44) in the main text. ρ plays a similar role as the level of decoherence of the first set of parameters. If $\rho = 1$, there is no mixing of wave packets and the subjective probability equals the objective probability, which corresponds to perfect rationality. If $\rho < 1$, the wave packets interfere with each other and can account for the difference between the subjective and objective probabilities. We examine the influence of parameters t, ρ and p_m by plotting the corresponding graphs for $\pi(t)$ vs p in Fig 1. The parameter ξ is kept at zero. We observe a linear dependence between the two variables. When ρ is too small, the map does not have a reasonable cognitive interpretation. Therefore, it is recommended to constrain the domain of ρ to some reasonable range (> 0.2, say). The graph shows a stronger overweighting at small objective probability p and underweighting at large p as t increases. The upper limit of the time effect is bounded by the value of ρ. From Fig 1, larger values of p_m cause the subjective probability to become increasingly independent from the objective one, with $\pi(p) = 0.5\ \forall p$ in the limit $p_m \to 1$. In agreement with empirical observations, the graphs show an overestimation and underestimation of small and large probabilities, respectively.

The third parameter choice uses different step sizes towards the left and right ends. The distance between the starting position to the left and the right ends are equal. The representation according to step sizes can replace the branch lengths in the formulation of a decision problem. The objective probability is given by $p = s_L/\left(s_L + s_R\right)$. The rest of the setup is the same as in the first case. Fig 2 shows that the dependence of $\pi(t)$ vs p exhibits an inverse double-S-shaped curve for different values of ρ. The level of decoherence tends to slow down the walk. The values of ρ act similarly to the first choice of parameters. However, the time variable does not change much the curves after a certain time, which means that the evaluation of probabilities saturates quickly.

The level of decoherence is controlled by the probability of performing a measurement at every position base. In this particular model, without introducing p_m, the continuous act of measurement at the boundaries already causes decoherence. Therefore, the probabilities will eventually lose coherence and become completely irreversible with time. This can be confirmed by observing that all curves eventually saturate to certain patterns over time. The loss of coherence with time either predicts a loss of oscillatory characteristics, or become a limitation of the model since there is no empirical observations confirming that a decision with a longer time contains less quantum-like behavior, such as hesitation that can be represented by oscillation of states. The problem of continuous measurements brings out a need for a coherent model.

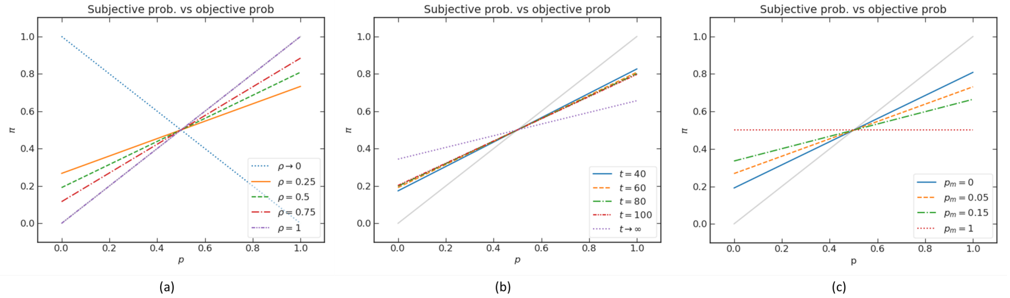

Fig 1. Subjective probability π vs objective probability p. The objective probability p is represented by the probability of obtaining a left spin in the initial state. The distance from x_0 to the two boundaries are equal and the distance between the two boundaries is kept as 50. The parameter ξ is set to zero. The parameters are (a) $t = 60$, varying ρ, $p_m = 0$. (b) varying t, $\rho = 0.5$, $p_m = 0$. (c) $t = 60$, $\rho = 0.5$, varying p_m.

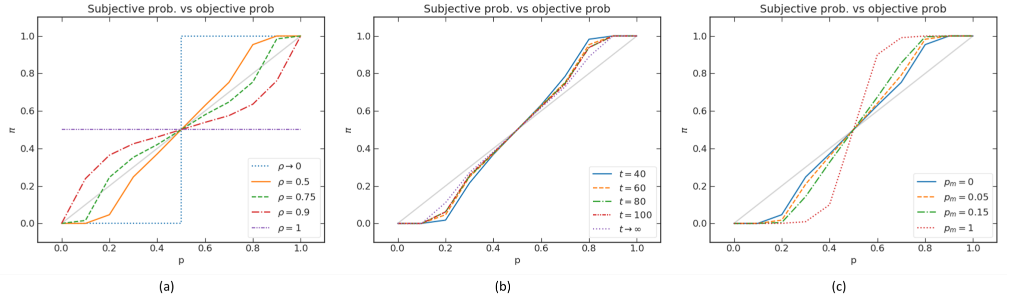

Fig 2. Subjective probability π vs objective probability p. The objective probability p is represented by the ratio of the left and right step lengths. The distance from x_0 to the two boundaries are equal and the distance between the two boundaries is kept as 50. We fix $\eta = 0.5$, $\xi = 0$. The other parameters as (a) $t = 60$, varying ρ, $p_m = 0$. (b) varying t, $\rho = 0.5$, $p_m = 0$. (c) $t = 60$, $\rho = 0.5$, varying p_m.
